# Pre-implantation genetic testing in ART: who will benefit and what is the evidence?

**DOI:** 10.1007/s10815-016-0785-2

**Published:** 2016-08-05

**Authors:** Alberto Vaiarelli, Danilo Cimadomo, Antonio Capalbo, Giovanna Orlando, Fabio Sapienza, Silvia Colamaria, Antonio Palagiano, Carlo Bulletti, Laura Rienzi, Filippo Maria Ubaldi

**Affiliations:** 1 G.EN.E.R.A. Centers for Reproductive Medicine, Rome, Italy; 2 GENETYX, Molecular Biology Laboratory, Marostica, Italy; 3 Dipartimento di Scienze Anatomiche, Istologiche, Medico Legali e dell’Apparato Locomotore, Sezione Istologia ed Embriologia Medica, University of Rome “Sapienza”, Rome, Italy; 4 Second University of Naples, Caserta, Italy; 5 Physiopathology of Reproduction Unit, Cattolica General Hospital, Cattolica, Italy

**Keywords:** PGD, PGD-A, Blastocyst, IVF, Aneuploidy testing, Counselling

## Abstract

Pre-implantation genetic diagnosis for aneuploidy testing (PGD-A) is a tool to identify euploid embryos during IVF. The suggested populations of patients that can benefit from it are infertile women of advanced maternal age, with a history of recurrent miscarriages and/or IVF failures. However, a general consensus has not yet been reached.After the clinical failure of its first version based on cleavage stage biopsy and 9 chromosome-FISH analysis, PGD-A is currently performed by 24 chromosome screening techniques on trophectoderm (TE) biopsies. This approach has been clearly demonstrated to involve a higher clinical efficiency with respect to the standard care, in terms of sustained pregnancy rate per transfer and lower miscarriage rate. However, data about PGD-A efficacy calculated on a per intention-to-treat basis, as well as an analysis of its cost-effectiveness, are still missing.TE biopsy is a safe and extensively validated approach with low biological and technical margin of error. Firstly, the prevalence of mosaic diploid/aneuploid blastocysts is estimated to be between 0 and 16 %, thus largely tolerable. Secondly, all the comprehensive chromosome screening (CCS) technologies adapted to, or designed to conduct PGD-A are highly concordant, and qPCR in particular has been proven to show the lowest false positive error rate (0.5 %) and a clinically recognizable error rate per blastocyst of just 0.21 %.In conclusion, there is a sufficient body of evidence to support the clinical application of CCS-based PGD-A on TE biopsies. The main limiting factor is the need for a high-standard laboratory to conduct blastocyst culture, biopsy and vitrification without impacting embryo viability.

## Introduction

Pre-implantation genetic diagnosis (PGD) in assisted reproductive technologies (ART) is suited to many inheritable genetic disorders caused by a known mutation and chromosome structural abnormalities, in order to increase the chance of delivering a healthy baby. However, the application of PGD is nowadays mainly focused on aneuploidy testing (PGD-A) in order to identify chromosomally normal embryos for the transfer. Since its first report in 1990 [1], PGD has always been considered a very intriguing but controversial topic, especially since PGD-A (or “PGS” as it has been referred to up to now) started to represent the vast majority of cases [2, 3].

The group of patients that have been suggested throughout the years to potentially benefit from PGD-A were infertile or sub-fertile women of *advanced maternal age* (AMA; usually, defined as ≥ 35 years), with a *history of recurrent pregnancy loss* (RPL; usually at least three previous miscarriages) or with *repeated implantation failure* (RIF; three or more failed embryo transfers) and *severe male factor* [4]. Over time, other indications have been proposed including a previous genetically abnormal pregnancy, poor embryo quality, previous radiotherapy and single embryo transfer (SET), even though a general international consensus has not been built yet.

Chromosomal aneuploidies are indeed the main reason for pregnancy loss and implantation failure, and their prevalence in the embryo is strongly related to female age [5, 6].

The ultimate goal of PGD-A is to reach the same efficacy of a standard IVF cycle, that is, the same number of babies born on a per intention-to-treat basis, meanwhile increasing the efficiency especially in terms of safety.

The aim of this review is to report “the state of the art” in the clinical validation and application of PGD-A.

## The failure of PGD-A in its first version

In 2011, Mastenbroek et al. [7] published a review and meta-analysis of all the randomized controlled trials (RCTs) published up to that date that compared aneuploidy testing versus standard IVF. They reported no beneficial effect of the former on the live birth rate. On the contrary, especially AMA patients were shown to have an even lower chance of conceiving when performing a PGD-A cycle. The authors proposed that some technical drawbacks could be the cause of such a failure and encouraged a careful pre-clinical validation of any novel PGD-A approach before its implementation in IVF. Such a negative outcome was ascribed to the strategy adopted at that time to conduct chromosomal analysis, namely single/double blastomere(s)-based 9 chromosome FISH analysis. Firstly, FISH analysis itself is highly limited since just 9 out of the 24 chromosomes composing the human karyotype were analyzed, which means that all the aneuploidies, which at that stage of embryo development can affect any chromosome, are ignored [8]. Secondly, the already low resolution of the techniques is also impacted by the pitfalls related to single-cell analysis. The combination of these two drawbacks led to high technical variability, over-estimation of mosaicism and an undiagnosed embryo rate as high as 10 % [9–12]. Thirdly, cleavage-stage biopsy strongly impacts embryo viability and its implantation potential, as Scott et al. well demonstrated in 2013 [13] in their randomized paired non-selection study. Specifically, they selected the two best quality embryos from a cohort produced by good prognosis women (defined as females younger than 35 years and with a normal ovarian reserve) during a standard IVF cycle, one was randomized to be biopsied while the other was left undisturbed. Both the embryos were then transferred without analysing the biopsied fragment. In case just one embryo implanted, then the fingerprinting data from the foetal DNA was compared to the DNA from the biopsied fragment in order to assess whether they corresponded or not. This design led the authors to define a 39 % significant relative reduction in implantation rate after cleavage-stage biopsy, which could then be classified as a detrimental approach. Interestingly, by applying the same study design to blastocyst stage biopsy, the authors reported no impact of this approach on embryo reproductive potential, since no difference in implantation rate was reported with respect to undisturbed blastocysts.

## The onset of PGD-A new version

A novel approach has been introduced after the several levels of pre-clinical validation, we will go through hereafter: TE biopsy at the blastocyst stage associated with comprehensive chromosome screening (CCS) techniques, such as array-Comparative Genomic Hybridization (aCGH), array-Single Nucleotide Polymorphisms (aSNP), quantitative Polymerase Chain Reaction (qPCR) and next-generation sequencing (NGS). Although, in 2014 Mastenbroek and Repping [14] published an opinion paper stating their concerns about a premature widespread application also of this novel strategy. Here, they drew attention to several points: the need to express PGD-A results on a per intention to treat basis rather than on a per embryo transfer basis, the need for unbiased and concordant RCTs, the importance of evaluating the cost-effectiveness of a PGD-A cycle, the estimation of the real prevalence of chromosomal mosaicism, as well as the duty to assess the sensitivity, specificity, positive and negative predictive values of any molecular method involved.

Hereafter, we will discuss the different types of evidence produced up to date (Table 1 represents a summary of the main published, as well as still missing evidences dealing with this topic).

**Table 1:** Levels of evidence of TE biopsy CCS-based PGD-A clinical value

Trophectoderm biopsy + comprehensive chromosome aneuploidy testing
Higher effectiveness with respect to the standard care
*Lee* et al.*, 2015, systematic review*
*Dahdouh* et al.*, 2015, meta-analysis*
*Chen* et al.*, 2015, meta-analysis*
Same efficacy as the standard care
*Ubaldi* et al.*,2015, Retrospective analysis*
*Still unproven by prospective or randomized controlled trials*
Higher cost-effectiveness with respect to the standard care
*Still never reported*
Tolerable prevalence of diploid/aneuploid blastocysts
*Capalbo* et al.*, 2013*; 70 blastocysts; no preferential allocation of aneuploid cells to the TE & 4 % diploid/aneuploid blastocysts
*Northrop* et al.*, 2010*; 50 blastocysts; no preferential allocation of aneuploid cells to the TE and 16 % diploid/aneuploid blastocysts
*Johnson* et al.*, 2010*; 51 blastocysts; no preferential allocation of aneuploid cells to the TE and 0 % diploid/aneuploid blastocysts
High positive and negative clinical predictive value
*Scott* et al.*, 2013, prospective randomised non-selection study (SNP-array)*
High concordance between CCS methods
*Capalbo* et al.*, 2016*, propective validation study
Very low clinically recognizable error rate
*Werner* et al.*, 2014 (qPCR)*
*Tiegs* et al.*, 2016 (aCGH)*

Summary of the main published papers and their relative take-home messages to support a safe clinical application of TE biopsy CCS-based PGD-A. Both the already existing, as well as the still missing, levels of evidence have been summarized in this Table. *ICM, Inner Cell Mass; TE, trophectoderm; CCS, Comprehensive Chromosome Screening*

## PGD-A effectiveness and efficacy

In a review published in 2015 [15], Lee and colleagues summarized all the RCTs, observational and prospective studies that approached CCS-based PGD-A in comparison to the standard care. They were able to show that in both young and AMA patient populations, PGD-A results in a higher delivery rate per embryo transferred. An equal clinical outcome can be determined only via double embryo transfer in the standard care group. To this regard, Forman and colleagues [16, 17] in particular showed that single euploid blastocyst transfer equals double untested embryo transfer in terms of live birth rate, but with significantly better obstetrical outcomes. In fact, they reported a lower birthweight and a longer period spent in the Neonatal Intensive Care Unit for new-borns from the standard care arm of the study. This was mainly a consequence of the establishment of multiple pregnancies in the untested double embryo transfer group, which were instead avoided by single euploid blastocyst transfers in the PGD-A group.

Dahdouh and colleagues and Chen and colleagues in 2015 published two meta-analyses [18, 19] of the RCTs and observational studies that adopted CCS-based PGD-A. They showed that this approach results in a higher sustained implantation rate per transfer, as well as lower miscarriage rate with respect to the control. These data collectively represent a considerable body of evidence in favour of PGD-A clinical efficiency, even though it should be highlighted that the RCTs published up to date were mostly performed in a good prognosis patient population.

It should be acknowledged though that the efficacy of PGD-A, namely the possibility to obtain the same delivery rate per intention to treat with respect to the standard care, is still to be demonstrated. Indeed, this is a critical aspect since it would prove that by extending the culture to the blastocyst stage, by performing TE biopsy and CCS, and by adopting a freeze-all and SET strategy, we are not causing any harm to the intrinsic reproductive potential of the embryo itself. To this regard, Ubaldi et al. in 2015 reported the clinical outcomes in AMA patient population across the years characterized by the gradual implementation of all these innovations in the clinical practice and by the sharp increase of the overall rate of the PGD-A cycles performed. Specifically, the cumulative live birth rate per cycle was kept constant across this period, while the miscarriage, and especially the multiple pregnancy rates significantly decreased [20]. This paper represented the first published evidence that the technical and procedural innovations in IVF (as PGD-A) do not impact its efficacy. Still though, the value of these data is strongly limited by the retrospective nature of the study. Multicentre RCTs comparing PGD-A to the standard care, whose primary outcome should be the delivery rate per intention to treat, are still eagerly needed in this field and are currently in the pipeline. Only the data produced through this study design can ultimately solve this still controversial issue.

At last, an analysis of the cost-effectiveness of PGD-A is also missing. Ideally, it should also take into account the obstetrical and neonatal costs, especially in the standard care arm of the study, where putatively higher miscarriage and multiple pregnancy rates would be registered. The cost analysis is obviously highly dependent on the molecular technique which is adopted for aneuploidy testing and from the regional costs of each type of medication, making such kinds of analysis of low widespread reproducibility. Regardless, it is of critical importance to provide comparative data about the cost-effectiveness of PGD-A in all the different settings in the future.

## Biological source of error

An important concern in PGD-A is chromosomal mosaicism, namely the presence in the same embryo of cells with different karyotypes due to a mitotic error occurring after fertilization. The earlier the mitotic error would occur along pre-implantation development, the higher would be its extent in the blastocyst. Its actual prevalence has still to be determined in embryos and several reports in literature may have over-estimated it, especially when based on single cell analysis at the cleavage stage with biased and inefficient techniques. Two concepts must be underlined: firstly, in a published dataset which entailed 5337 consecutive chorionic villus samplings, there was no difference in the prevalence of mosaicism at the end of the first trimester in pregnancies conceived spontaneously compared to those from infertile couples. Furthermore, there was no difference either in the prevalence of mosaicism when in vitro (1.32 %) and in vivo treatments (1.22 %) were compared in infertile couples, where the reported mosaicism rate confirmed in a follow-up amniocentesis analysis was only 0.3–0.44 % [21]. Secondly, mosaicism will always represent a source of biological variability where the analysis on a randomly selected TE sample from a diploid/aneuploid blastocyst (the only one at risk of misdiagnosis) cannot mirror the actual chromosomal constitution of the whole embryo.

People in the field were also concerned about the hypothesis of a preferential allocation of aneuploid cells to the TE. There are mainly three papers in literature that investigated this hypothesis: in 2013, Capalbo et al. described a method to biopsy the inner cell mass (ICM) that ensured 85.7 % of samples with no TE cells contamination, and a contamination level lower than 2 % in the rest [22]. Seventy blastocysts previously analysed by aCGH were warmed and underwent to ICM biopsy as well as to the retrieval of 3 more TE fragments which were then all analysed by FISH. No preferential allocation of aneuploid cells to the TE was reported. In 2010, Northrop et al. performed the aSNP-based blastocyst stage re-analysis of the ICM and 3 TE fragments from 50 embryos that were previously given an aneuploid diagnosis by cleavage stage FISH [23]. Also here, there was no report of a preferential allocation of aneuploid cells to the TE. In the same year, Johnson et al. had also performed a similar analysis by aCGH on the ICM and 2TE fragments from 51 blastocysts, again reporting no preferential allocation [24]. Furthermore, in these 3 papers, the prevalence of mosaic diploid/aneuploid blastocysts can be collectively estimated around 5 %, thus largely tolerable.

Recently, Greco et al. reported to have transferred presumably mosaic blastocysts diagnosed by aCGH to consenting patients after counselling [25]. The authors stated that they were able to identify 181 mosaic blastocysts out of 3802 analysed (4.8 %), among which 18 were transferred to achieve 6 full-term healthy pregnancies. This outcome suggests a scenario in which mosaic blastocyst still does have the possibility to result in a clinical pregnancy; however, the reliability of the molecular analysis adopted in this study is somehow questionable and the diagnosis of mosaicism can be partially ascribed to false positive errors of aCGH, but we will discuss this issue in the next paragraph.

In a previous prospective randomised non-selection study by Scott and colleagues [26], the authors already reported the data derived from the SET of blastocysts that underwent the biopsy of a TE fragment which was analysed only after the definition of the clinical outcome downstream. In particular, the clinical negative predictive value (the rate of CCS-diagnosed aneuploid blastocysts that failed to implant) of TE-based PGD-A through aSNP technology was reported as 93.5 %, while the positive predictive value (the rate of CCS-diagnosed euploid blastocysts that resulted in a sustained implantation) was reported as 48.2 %. Recently, the same group has presented the data produced with the same design but after a targeted NGS analysis-based approach (no whole genome amplification involved) [27]. Importantly, 100 % of the blastocysts that were assigned an aneuploid diagnosis (*n* = 41) did not actually implant in their interim analysis.

In conclusion, although real mosaicism and methodological aspects can impact the reliability of the diagnosis due to false positive errors, up to date, there has been no evidence at all from well-designed pre-clinical studies that this is a major issue in the application of PGD-A as long as validated technologies are used for CCS.

## Technical source of error

Every time a technique or embryo selection parameter is introduced in clinical practice, it is pivotal to perform a validation, as well as an analysis of the level of accuracy in terms of specificity and sensitivity. Both the advantages and the limitations of this should be clearly expressed to the patients (). To this regard, all the CCS methods adapted to or designed to perform PGD-A showed very high consistency in the detection of whole chromosome imbalances when compared in the blinded validation study performed by Capalbo et al. and published in 2015 [28]. However, aCGH was characterized in this study by a 7 % false-positive error rate (0.5 % in the case of qPCR), which may be a consequence of the artefacts derived by the use of whole genome amplification (WGA). This approach in fact entails a random amplification of the genome which can result in an uneven amount of the DNA sequences at some genomic locations and consequently in technical artefacts. Such a pitfall is instead prevented by targeted amplification protocols that are limited to non-variable regions in the genome, as the ones that characterize qPCR or targeted NGS [29, 30]. Obviously, these last approaches result in a lower resolution since structural imbalances and/or partial aneuploidies cannot be detected (array-based or WGA-based NGS approaches can), but they guarantee an accuracy on embryo biopsies higher than 99 %. Importantly, a validation on cell lines of known karyotype has been performed for both aSNP and qPCR [28, 29, 31] and is instead still missing for aCGH, despite this should be the very first level of validation for any novel technique. A provocative consideration is the possibility that not all the diagnoses of segmental aneuploidies or mosaicism in studies that adopted WGA-based technologies can be reliable and actually mirror the real chromosomal status of the embryo. Rather, they can be an artefact of WGA, which should be acknowledged by the providers. In our opinion, it is dangerous to produce some data which still cannot be interpreted with a sufficiently high level of accuracy, since it can affect the overall efficacy of PGD-A as it occurred for its first version.

In 2014, Werner et al. [32] published the data describing the clinically recognizable error rate after qPCR-based CCS. Here they showed a largely tolerable 0.21 % (10/4794) error rate per blastocyst. In particular, 7 errors were found in the product of conception (4 were due to real chromosomal mosaicism) and 3 resulted in a full-term delivery. In contrast, in 2016, Tiegs et al. reported a 0.9 % (5/579) error rate per blastocyst analysed after aCGH-based PGD-A [33]. Similar reports are still eagerly needed for all the other molecular techniques adopted for PGD-A.

## Indications to PGD-A and guidelines according to the international societies

The Canadian Society of Obstetrics and Gynaecology encouraged not to perform the PGD-A strategy entailing the FISH-based analysis of blastomeres in favour of the CCS-based analysis of TE biopsies. The latter is in fact reported here as the gold standard approach due to the favourable clinical outcomes it ensures [34]. The authors endorsed PGD-A application particularly for good prognosis patients since it may result in an improvement with respect to the standard criteria for embryo selection.

The US Assisted Reproductive Technology Surveillance Data of years 2011–2012 were published in 2016 [35]. Here, the authors support the higher safety of PGD-A with respect to the standard care in terms of lower odds of miscarriage and, for patients older than 37, the higher likelihood of achieving a live-birth delivery per transfer. To this regard, PGD-A was strongly recommended for patients aged older than37.

What the indications to PGD-A should be is still a controversial topic. It is generally indicated for AMA patients, even though a threshold of age has not been defined yet. Franasiak et al. [36] reported on more than 15,000 CCS-screened TE biopsies an exponential increase in the aneuploidy rate from 35 years of maternal age onwards, which rises to 90 % by the age of 44. Based on this data, most of the blastocysts obtained in this patient population are aneuploid and thus not reproductively competent. The identification of the euploid blastocysts is a pivotal tool because we can avoid useless and potentially dangerous transfers. To this end, the tailoring of ovarian stimulation is crucial and novel strategies such as double stimulation in a single menstrual cycle (Duostim) [37] in poor prognosis patients could result in a higher possibility to obtain an euploid embryo in the shortest possible time.

Finally, Franasiak et al. [36] also reported a 30 % baseline production of aneuploid embryos in very young patients (<35 years). This evidence suggests the possibility to extend PGD-A application to good prognosis patients who produce a high number of blastocysts, especially if they have an history of previous IVF failures (RIF) and/or miscarriages (RPL). This can reduce the number of blastocysts candidate to the transfer and support a SET policy also in this patient population. Furthermore, the systematic application of PGD-A in allegedly good prognosis patients holds the potential to identify a subgroup of patients that produce a significantly higher rate of aneuploid embryos than what is expected on the basis of female age only. This is extremely important in a context where no biomarker is yet available to predict such an outcome.

At last, RCTs that investigate the value of PGD-A in purely RPL and purely RIF patients are also missing. They are eagerly needed though, in order to assess whether aneuploidy screening can increase the efficiency of IVF also in such a poor prognosis population, where maternally derived embryonic aneuploidies eventually are not the main cause of infertility.

## Conclusions

Nowadays and ever since PGD-A was introduced in the clinical practice, the counselling given to a couple has changed dramatically. In particular, the primary aim now is not to perform the transfer of an excellent/good quality embryo anymore, especially since the morphological quality is only mildly correlated with euploidy rate [8], but to identify a blastocyst with a high reproductive potential. PGD-A is a tool which can enhance our predictive power of the clinical outcomes because, once an euploid blastocyst is obtained, the patient is provided with a ≈ 50 % sustained implantation potential across the border of female age and independently from embryo morphological/morphodynamic quality. Good prognosis patients who produce many blastocysts can also then benefit from aneuploidy screening since it could potentially shorten the time required to perform the transfer of the competent embryo(s).

A high-standard laboratory is required to successfully implement a PGD-A programme. In particular, we should be able to guarantee a proper culture system to maximize blastocyst rate, proper embryo grading parameters so as not to discard viable embryos, an efficient vitrification technique to minimize degeneration after warming, skilled embryologists to conduct TE-based biopsy and proper CCS techniques with the lowest possible no amplification, false-positive and negative error rates. All these aspects are essential so as not to reduce the efficacy of IVF and, overall, they represent the main limiting factor for an extensive application of PGD-A worldwide.

## References

[1] Handyside AH, Kontogianni EH, Hardy K, Winston RM (1990). Pregnancies from biopsied human preimplantation embryos sexed by Y-specific DNA amplification. Nature.

[2] Coonen E, De Rycke M, Kokkali G (2015). Data from the ESHRE PGD Consortium 2015. ESHRE Abstract Book, Annual Meeting.

[3] Moutou C, Goossens V, Coonen E, De Rycke M, Kokkali G, Renwick P (2014). ESHRE PGD Consortium data collection XII: cycles from January to December 2009 with pregnancy follow-up to October 2010. Hum Reprod.

[4] Harper JC, Boelaert K, Geraedts J, Harton G, Kearns WG, Moutou C (2006). ESHRE PGD Consortium data collection V: cycles from January to December 2002 with pregnancy follow-up to October 2003. Hum Reprod.

[5] Nagaoka SI, Hassold TJ, Hunt PA (2012). Human aneuploidy: mechanisms and new insights into an age-old problem. Nat Rev Genet.

[6] Hassold T, Hunt P (2001). To err (meiotically) is human: the genesis of human aneuploidy. Nat Rev Genet.

[7] Mastenbroek S, Twisk M, van der Veen F, Repping S (2011). Preimplantation genetic screening: a systematic review and meta-analysis of RCTs. Hum Reprod Update.

[8] Capalbo A, Rienzi L, Cimadomo D, Maggiulli R, Elliott T, Wright G (2014). Correlation between standard blastocyst morphology, euploidy and implantation: an observational study in two centers involving 956 screened blastocysts. Hum Reprod.

[9] Treff NR, Scott RT (2012). Methods for comprehensive chromosome screening of oocytes and embryos: capabilities, limitations, and evidence of validity. J Assist Reprod Genet.

[10] Voullaire L, Slater H, Williamson R, Wilton L (2000). Chromosome analysis of blastomeres from human embryos by using comparative genomic hybridization. Hum Genet.

[11] Wells D, Delhanty JD (2000). Comprehensive chromosomal analysis of human preimplantation embryos using whole genome amplification and single cell comparative genomic hybridization. Mol Hum Reprod.

[12] Mertzanidou A, Wilton L, Cheng J, Spits C, Vanneste E, Moreau Y (2013). Microarray analysis reveals abnormal chromosomal complements in over 70% of 14 normally developing human embryos. Hum Reprod.

[13] Scott RT, Upham KM, Forman EJ, Zhao T, Treff NR (2013). Cleavage-stage biopsy significantly impairs human embryonic implantation potential while blastocyst biopsy does not: a randomized and paired clinical trial. Fertil Steril.

[14] Mastenbroek S, Repping S (2014). Preimplantation genetic screening: back to the future. Hum Reprod.

[15] Lee E, Illingworth P, Wilton L, Chambers GM (2015). The clinical effectiveness of preimplantation genetic diagnosis for aneuploidy in all 24 chromosomes (PGD-A): systematic review. Hum Reprod.

[16] Forman EJ, Hong KH, Ferry KM, Tao X, Taylor D, Levy B (2013). In vitro fertilization with single euploid blastocyst transfer: a randomized controlled trial. Fertil Steril.

[17] Forman EJ, Hong KH, Franasiak JM, Scott RT (2014). Obstetrical and neonatal outcomes from the BEST Trial: single embryo transfer with aneuploidy screening improves outcomes after in vitro fertilization without compromising delivery rates. Am J Obstet Gynecol.

[18] Dahdouh EM, Balayla J, Garcia-Velasco JA (2015). Comprehensive chromosome screening improves embryo selection: a meta-analysis. Fertil Steril.

[19] Chen M, Wei S, Hu J, Quan S (2015). Can comprehensive chromosome screening technology improve IVF/ICSI outcomes? a meta-analysis. PLoS One.

[20] Ubaldi FM, Capalbo A, Colamaria S, Ferrero S, Maggiulli R, Vajta G (2015). Reduction of multiple pregnancies in the advanced maternal age population after implementation of an elective single embryo transfer policy coupled with enhanced embryo selection: pre- and post-intervention study. Hum Reprod.

[21] Huang A, Adusumalli J, Patel S, Liem J, Williams J, Pisarska MD (2009). Prevalence of chromosomal mosaicism in pregnancies from couples with infertility. Fertil Steril.

[22] Capalbo A, Wright G, Elliott T, Ubaldi FM, Rienzi L, Nagy ZP (2013). FISH reanalysis of inner cell mass and trophectoderm samples of previously array-CGH screened blastocysts shows high accuracy of diagnosis and no major diagnostic impact of mosaicism at the blastocyst stage. Hum Reprod.

[23] Northrop LE, Treff NR, Levy B, Scott RT (2010). SNP microarray-based 24 chromosome aneuploidy screening demonstrates that cleavage-stage FISH poorly predicts aneuploidy in embryos that develop to morphologically normal blastocysts. Mol Hum Reprod.

[24] Johnson DS, Cinnioglu C, Ross R, Filby A, Gemelos G, Hill M (2010). Comprehensive analysis of karyotypic mosaicism between trophectoderm and inner cell mass. Mol Hum Reprod.

[25] Greco E, Minasi MG, Fiorentino F (2015). Healthy babies after intrauterine transfer of mosaic aneuploid blastocysts. N Engl J Med.

[26] Scott RT, Ferry K, Su J, Tao X, Scott K, Treff NR (2012). Comprehensive chromosome screening is highly predictive of the reproductive potential of human embryos: a prospective, blinded, nonselection study. Fertil Steril.

[27] Werner MD, Franasiak JM, Hong KH, Juneau CR, Tao X, Landis J, Upham KM, Treff NR, Scott RT. A prospective, blinded, non-selection study to determine the predictive value of ploidy results using a novel method of targeted amplification based Next generation sequencing (NGS) for comprehensive chromosome screening (CCS). ASRM abstract book 2015.

[28] Capalbo A, Treff NR, Cimadomo D, Tao X, Upham K, Ubaldi FM (2015). Comparison of array comparative genomic hybridization and quantitative real-time PCR-based aneuploidy screening of blastocyst biopsies. Eur J Hum Genet.

[29] Treff NR, Tao X, Ferry KM, Su J, Taylor D, Scott RT (2012). Development and validation of an accurate quantitative real-time polymerase chain reaction-based assay for human blastocyst comprehensive chromosomal aneuploidy screening. Fertil Steril.

[30] Treff NR, Fedick A, Tao X, Devkota B, Taylor D, Scott RT (2013). Evaluation of targeted next-generation sequencing-based preimplantation genetic diagnosis of monogenic disease. Fertil Steril.

[31] Treff NR, Su J, Tao X, Levy B, Scott RT (2010). Accurate single cell 24 chromosome aneuploidy screening using whole genome amplification and single nucleotide polymorphism microarrays. Fertil Steril.

[32] Werner MD, Leondires MP, Schoolcraft WB, Miller BT, Copperman AB, Robins ED (2014). Clinically recognizable error rate after the transfer of comprehensive chromosomal screened euploid embryos is low. Fertil Steril.

[33] Tiegs AW, Hodes-Wertz B, McCulloh DH, Munné S, Grifo JA (2016). Discrepant diagnosis rate of array comparative genomic hybridization in thawed euploid blastocysts. J Assist Reprod Genet.

[34] Dahdouh EM, Balayla J, Audibert F, Genetics C, Wilson RD, Audibert F (2015). Technical update: preimplantation genetic diagnosis and screening. J Obstet Gynaecol Can.

[35] Chang J, Boulet SL, Jeng G, Flowers L, Kissin DM (2016). Outcomes of in vitro fertilization with preimplantation genetic diagnosis: an analysis of the United States Assisted Reproductive Technology Surveillance Data, 2011–2012. Fertil Steril.

[36] Franasiak JM, Forman EJ, Hong KH, Werner MD, Upham KM, Treff NR (2014). The nature of aneuploidy with increasing age of the female partner: a review of 15,169 consecutive trophectoderm biopsies evaluated with comprehensive chromosomal screening. Fertil Steril.

[37] Ubaldi FM, Capalbo A, Vaiarelli A, Cimadomo D, Colamaria S, Alviggi C (2016). Follicular versus luteal phase ovarian stimulation during the same menstrual cycle (DuoStim) in a reduced ovarian reserve population results in a similar euploid blastocyst formation rate: new insight in ovarian reserve exploitation. Fertil Steril.

